# 16-year excess all-cause mortality of newly diagnosed type 2 diabetic patients: a cohort study

**DOI:** 10.1186/1471-2458-9-400

**Published:** 2009-10-31

**Authors:** Lars J Hansen, Niels de Fine Olivarius, Volkert Siersma

**Affiliations:** 1 The Research Unit for General Practice and Section of General Practice, Department of Public Health, University of Copenhagen, Copenhagen, Denmark

## Abstract

**Background:**

Studies have shown that type 2 diabetic patients have higher all-cause mortality than people without diabetes, but it is less clear how diabetes affects mortality in elderly patients and to what degree mortality differs between diabetic men and women. The aim of the present study is to investigate the age- and sex-specific all-cause mortality pattern in patients with type 2 diabetes in comparison with the Danish background population.

**Methods:**

Population-based cohort study of 1323 patients, diagnosed with clinical type 2 diabetes in 1989-92 and followed for 16 years. Median (interquartile range) age at diagnosis was 65.3 (55.8-73.6) years. The age- and sex-specific hazard rates were estimated for the cohort using the life table method and compared with the expected hazard rates calculated with Danish register data from the general population.

**Results:**

In comparison with the general population, diabetic patients had a 1.5-2.5 fold higher risk of dying depending on age. The over-mortality was higher for men than for women. It decreased with age in both sexes, and among patients over 80 years at diagnosis the difference between the observed and the expected survival was small.

**Conclusion:**

We found an excess mortality of type 2 diabetic patients compared with the background population in all age groups. The excess mortality was most pronounced in men and in young patients.

## Background

Studies have shown that type 2 diabetic patients have higher all-cause mortality than people without diabetes [1-12], but it is less clear how diabetes affects mortality in elderly patients [13] and to what degree mortality differs between diabetic men and women [13,14]. An increased mortality is commonly reported in diabetic patients under the age of 70 years at diagnosis [2,3] and in some studies also in patients over this age [1,6,7,9]. Previous research, however, found no increased mortality in patients over 70 years of age irrespective of sex [5] or in elderly male patients [2,3,15] or elderly female patients [10]. This may partly be explained by short follow-up times and few participants which may reflect lack of power to detect a real effect of patient's sex and age on mortality [2,5,10,15]. Furthermore, a sex-related difference in all-cause mortality may have changed over time [14] and possibly to a different degree in different countries [16].

This study presents the long-term follow-up of a Danish population-based cohort of 1323 newly diagnosed type 2 diabetic patients aged 40 years or over [10]. The aim of the present study was to investigate the mortality patterns over the first 16 years after diabetes diagnosis in comparison with the general Danish population, and to assess the influence of age and sex.

## Methods

At the end of 1988, 474 general practitioners (GPs) volunteered to take part in the Danish study, Diabetes Care in General Practice [17]. Before the study started, the GPs were randomized to an intervention and a comparison group. All doctors were to include all patients on their practice list fulfilling the following criteria: 1) diabetes mellitus diagnosed from 1 March 1989 to 28 February 1991 (71 GPs volunteered for a 1-year extension of this period to establish a large cohort of patients suitable for epidemiological research), 2) based on hyperglycaemic symptoms and/or raised blood glucose values measured in general practice, the diagnosis was established by a single fasting whole blood or plasma glucose concentration ≥7.0/8.0 mmol/l measured at a major laboratory, and 3) age 40 years or older at diagnosis. The GPs were repeatedly instructed not to alter diagnostic practice during the inclusion period and to include all newly diagnosed patients. Patients who were in hospital at the time of diagnosis were also considered for inclusion in the study at their first visit to their GP after the diagnosis. When the inclusion period ended we asked the GPs how many individuals they had not included. Their responses revealed that 90 patients had been diagnosed but not considered for inclusion in the trial and, of these, 40 patients would eventually have been included if the GPs had remembered or managed to do so. The inclusion activity was similar in the two groups of doctors and it did not change over time [17].

There were 358,912 subjects aged 40 years or over on the list of patients in the participating practices by 1 March 1991 (More than 97% of the Danish population are registered with a GP in a tax-based, health insurance system) [18]. A total of 1543 newly diagnosed diabetic patients were eligible, but 162 were excluded because of protocol-based exclusion criteria: life threatening somatic disease (50), severe mental illness (50), or unwillingness to participate (62). In addition, 46 patients treated with steroids and 12 non-Caucasian patients were excluded from the present analysis, giving the final sample of 1323 patients. According to start of insulin treatment within 180 days of diagnosis, 97.6% of the patients were considered to have type 2 diabetes [17].

The vital status of all the diabetic patients was certified on the 31 January 2006 through The Danish Civil Registration System . This is the main administrative registry in Denmark, which includes complete and continuously updated information on all Danish residents on vital status: date of birth, and whether the person is alive, deceased or has emigrated, along with the dates of these events [19]. Among our cohort of patients, the vital status for one patient could not be assessed because this person had emigrated in 1992.

The remaining variables in Table 1 have been described elsewhere [17].

**Table 1:** Baseline characteristics. Values are medians (interquartile ranges) or number (%)

**Variable**	**Men** **n = 704**	**Women** **n = 619**	**Total** **n = 1323**
Age at diagnosis (years)	63.6 (54.1-71.4)	67.5 (57.8-75.4)	65.3 (55.8-73.6)

Diagnostic plasma glucose (mmol/l)	13.7 (10.8-16.9)	13.7 (10.5-17.0)	13.7 (10.7-17.0)

Total cholesterol (mmol/l)	6.1 (5.2-6.9)	6.3 (5.6-7.3)	6.2 (5.4-7.1)

Triglyceride (mmol/l)	1.96 (1.38-3.01)	1.97 (1.42-2.85)	1.96 (1.40-2.91)

Systolic blood pressure (mm Hg)	144 (130-160)	155 (140-170)	150 (130-160)

Body mass index (kg/m^2^)	29.0 (26.3-31.7)	29.6 (26.0-33.7)	29.2 (26.2-32.8)

Urinary albumin (mg/ml)	13.3 (7.0-36.0)	9.7 (4.8-21.1)	11.6 (5.8-29.1)

Cardiovascular disease	214 (31.3)	178 (29.4)	392 (30.4)

Diabetic retinopathy	33 (5.3)	23 (4.2)	56 (4.8)

Peripheral neuropathy	139 (20.0)	112 (18.4)	251 (19.3)

Known cancer	26 (3.8)	51 (8.4)	77 (6.0)

Smoking			

Never	90 (13.2)	295 (48.8)	385 (29.9)

Former	299 (43.8)	147 (24.3)	446 (34.6)

Current	294 (43.0)	163 (26.9)	457 (35.5)

Physical activity			

Low	158 (23.2)	191 (31.5)	349 (27.1)

Moderate	455 (66.9)	396 (65.2)	851 (66.1)

High	67 (9.9)	20 (3.3)	87 (6.8)

The protocol was approved by the ethics committee of Copenhagen and Frederiksberg.

### Statistical analysis

Patients randomized to the intervention and the comparison group were analysed together since the intervention was not a significant predictor of mortality [17]. Survival functions were estimated by a Kaplan-Meier estimator: gender differences were tested by log-rank tests. Sex- and age-specific (age in 5-year intervals) hazard rates for mortality in the general Danish population were calculated from national register data from the years 2001-2005 and 1991-1995 (., data accessed June 2007) as the average of the age-specific death intensities for the ages in the age-group. For the cohort of diabetic patients corresponding hazard rates were estimated using the life table method [20], i.e. a constant death intensity was estimated in each age and sex group as the number of deaths in the group divided by the total time lived in the group; and patients moved from one age group to the next as they became older. Approximate 95% confidence intervals were calculated using a normal distribution for the logarithm of the estimated death intensities. The yearly survival probability over the first 16 years after diabetes diagnosis thus estimated for the diabetic patients was compared with the expected survival probability calculated for a sample of the general population with same gender and age distribution as the cohort of diabetic patients.

## Results

**Figure 1:**
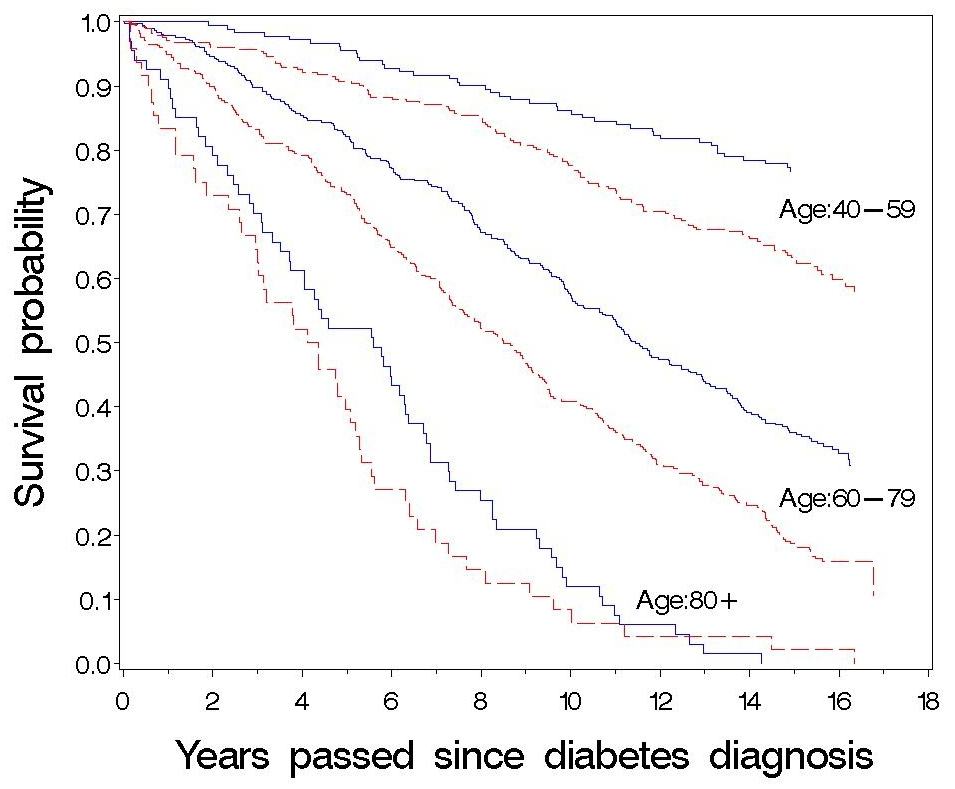
**Survival of diabetic patients after diabetes diagnosis according to gender and age at diagnosis**. Kaplan-Meier curves. Males: ------ (red). Females: — (blue). Log-rank test, p = 0.0003 (age 40-59 years), p < 0.0001 (age 60-79 years), p = 0.2984 (age ≥80 years).

During the follow-up period of median (interquartile range) 15.9 (15.3-16.4) years more men (67.1%) than women (57.5%) died (P = 0.0003). At diagnosis, women had higher age, higher systolic blood pressure, less urinary albumin and more favourable smoking habits than men, but otherwise the two sexes differed little concerning risk factors and diabetic complications (Table 1). The excess mortality in men compared with woman was seen in all age groups (Table 2) and seems fairly constant over time after diagnosis (Fig. 1).

**Table 2:** Number of deaths from diabetes diagnosis until 31 January 2005

	**Males**				**Females**			
**Age at Diabetes Diagnosis (years)**	**No. of Subjects**	**Person-Years of Observation**	**No. of Deaths**	**%**	**No. of Subjects**	**Person-Years of Observation**	**No. of Deaths**	**%**

40-44	37	520	8	21.6	26	409	1	3.9

45-49	73	1006	19	26.0	35	532	3	8.6

50-54	82	1045	35	42.7	61	844	16	26.2

55-59	89	1071	49	55.1	59	782	22	37.3

60-64	108	1089	73	67.6	79	1071	28	35.4

65-69	112	1055	91	81.3	96	1088	60	62.5

70-74	96	766	93	96.9	99	1037	76	76.8

75-79	59	381	56	94.9	97	778	83	85.6

80-85	35	169	35	100	47	278	47	100

85+	13	52	13	100	20	92	20	100

Total	704	7154	472	67.1	619	6910	356	57.5

**Figure 2:**
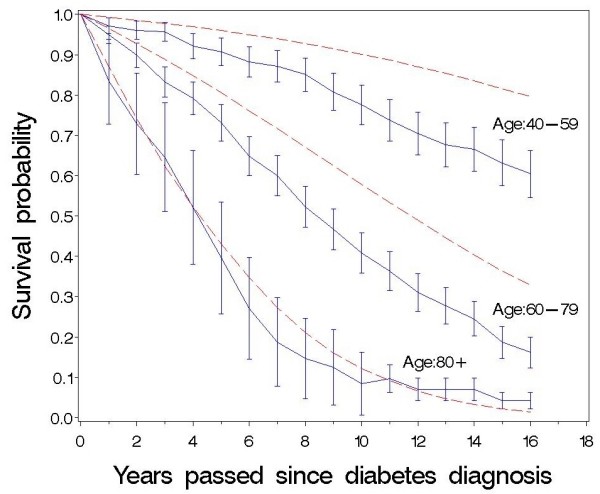
**Observed and expected survival curve of newly-diagnosed diabetic patients up to 16 years after diabetes diagnosis**. Men, life table method, diabetic patients: — (blue), 95% confidence intervals shown, Danish population: ------ (red).

**Figure 3:**
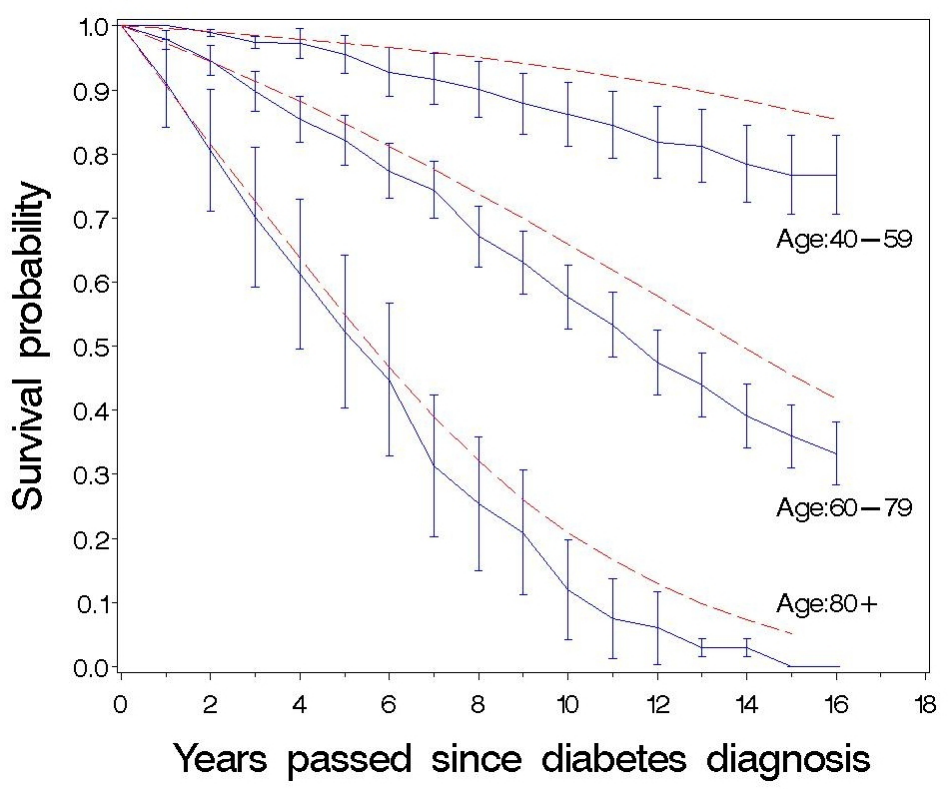
**Observed and expected survival curve of newly-diagnosed diabetic patients up to 16 years after diabetes diagnosis**. Females, life table method, diabetic patients: — (blue), 95% confidence intervals shown, Danish population: ------ (red).

In Fig. 2 and 3 the observed and expected survival are depicted by applying the life table method, as proposed by Lee [20]. Fig. 2 shows that the survival curve for the male diabetic patients aged 40-79 years separates from the corresponding curve for the Danish population immediately after diabetes diagnosis. This happens only after 3-5 years for women in the same age group (Fig. 3).

Compared with the Danish population, the diabetic patients had an increased mortality risk during the 16 years of follow-up (Table 3, Fig. 2 and 3) and males seem to have a slightly higher over-mortality than women (Table 3).

**Table 3:** Hazard rates during the first 16 years after diabetes diagnosis as compared with the Danish Population

**Age at Time of Death (years)**	**Diabetic Patients**			**Danish population**		**Relative risk (2001-05) ‡**	**Relative risk (1991-95) λ**
				
	**No. of Deaths**	Hazard Rate*	**(95% Confidence Interval)**	**Hazard rate (2001-05)†**	**Hazard rate (1991-95)φ**		
**Males**							

40-44	3	40.0	(12.9-124.0)	2.3	3.0	17.7	14.4

45-49	3	9.0	(2.9-28.0)	3.8	4.3	2.4	2.1

50-54	11	15.6	(8.7-28.2)	6.0	6.7	2.6	2.3

55-59	23	22.7	(15.1-34.1)	9.0	11.5	2.5	2.0

60-64	45	39.4	(29.4-52.8)	14.2	19.2	2.8	2.1

65-69	64	54.8	(42.9-70.1)	23.3	30.6	2.4	1.8

70-74	77	69.2	(55.3-86.5)	38.2	48.6	1.8	1.4

75-79	85	95.0	(76.8-117.5)	62.6	74.3	1.5	1.3

80-85	92	184.1	(150.1-225.8)	101.5	113.1	1.8	1.6

85+§	52	265.9	(202.7-349.0)				

Total	455	63.7	(58.1-69.9)				

**Females**							

40-44	0	0.0	(0.0-0.4)	1.3	1.8	0.0	0.0

45-49	0	0.0	(0.0-0.1)	2.4	2.9	0.0	0.0

50-54	4	9.3	(3.5-24.9)	3.8	4.7	2.5	2.0

55-59	10	14.5	(7.8-27.0)	5.8	7.6	2.5	1.9

60-64	13	15.3	(8.9-26.3)	9.2	12.3	1.7	1.3

65-69	22	21.9	(14.4-33.2)	15.4	18.5	1.4	1.2

70-74	41	36.9	(27.2-50.1)	25.8	28.4	1.4	1.3

75-79	68	60.0	(47.3-76.1)	40.6	44.3	1.5	1.4

80-85	86	95.7	(77.5-118.2)	67.2	74.9	1.4	1.3

85+§	75	171.6	(136.9-215.2)				

Total	319	46.7	(41.8-52.1)				

*Hazard rates (×1000) estimated as number of deaths divided by person years of observation.

†Hazard rates (×1000) estimated as average mortality rates from the life table 2001-2005 of the Danish population.

φ Hazard rates (×1000) estimated as average mortality rates from the life table 1991-1995 of the Danish population

‡This is calculated by dividing the hazard rate of diabetic patients by the hazard rate of the Danish population (2001-2005).

λ This is calculated by dividing the hazard rate of diabetic patients by the hazard rate of the Danish population (1991-1995).

§Among subjects aged 85 years or over, the age distribution for diabetic patients is very different from that of the background population. Therefore, a comparable hazard rate for the background population cannot be calculated

The excess mortality in the cohort of patients compared with the Danish population decreases with increasing age in both sexes (Table 3), and among patients 80 years or over at diagnosis the difference between the observed and the expected survival is small (Fig. 2 and 3). As can be seen in table 3, the relative risks are a little lower in all age groups if we use hazard rates for mortality in the general Danish population from the years 1991-1995 instead of 2001-2005, but our finding that the relative risk decreases with age persists.

## Discussion

In this population-based study of 1323 primarily type 2 diabetic patients followed for 16 years from the day of diagnosis, we found an excess mortality compared with the background population in all age groups. The over-mortality generally seemed to be higher for males than for females and it decreased with age in both sexes.

The list-patient system in Danish general practice with a well-defined background population in each practice, the unchanged inclusion activity during the inclusion period, the few exclusions and doctors' self-reports suggest that patients in this study are likely to be a representative sample of patients aged 40 years of age or over with newly diagnosed clinical diabetes in Denmark. Based on the information given by the GPs, only about 5% of eligible cases with newly diagnosed diabetes were not considered for inclusion or primary exclusions. A relatively high proportion of the oldest patients die shortly after diabetes diagnosis, but this early mortality could have been even greater if the GPs had included the 50 patients excluded because of severe somatic disease. Many of these patients were sick and dying, and the decision to exclude them was made by the GPs mainly following ethical considerations. Furthermore, the long-term follow up during which almost 2/3 of the patients died strengthens the results in this paper. Moreover, in comparison with other studies that included patients with different diabetes duration [21], we included only newly diagnosed patients which minimizes the risk of selecting an artificially healthy cohort of diabetic patients [22]. Finally, we used data from the Danish Civil Register which enabled collection of almost complete information on mortality over the 16-year follow-up period. Vital status was only unknown for one person, who emigrated.

It is a limitation that the reference population encompassed diabetic patients. This may have underestimated the observed excess mortality especially among the oldest patients where diabetes prevalence is highest (i.e. between 10-15%). We did not adjust for established risk factors for death since this information was not available from the reference population. Other studies, adjusting for such risk factors, have found that the risk of death among people with diabetes relative to those without diabetes is generally equal to or only slightly lower than the crude age-adjusted estimate of the relative risk [14,23,24].

In accordance with mortality studies from The United Kingdom [1,6,7], Italy [2], Germany [3], The Netherlands [4], The United States [14], Brazil [9] and Australia [5] we found that type 2 diabetic patients in comparison with the general population had a 1.5-2.5 fold higher risk of mortality depending on the age of the patients. An excess risk of death has previously been found even for newly diagnosed diabetic patients in population-based studies [3,25].

The survival curve for diabetic men aged 40-79 years separates from the corresponding curve for the Danish population immediately after diabetes diagnosis whereas this happens after approximately 6 years for women of the same age showing that both female and male diabetic patients have increased mortality risk compared with the general population (Fig. 2 and 3). Our study, however, indicates that in most age groups diabetic men have higher excess risks than diabetic women (Table 3). This is in accordance with results from Poland [26] and Japan [27]. Recent results from the US [14] indicate that reduction in mortality rates among diabetic men has reduced the previously observed female-over-male advantage in mortality among diabetic patients, and the sex-difference in mortality we observed may also have lessened in recent years due to a faster decrease in the mortality rate among Danish male diabetic patients relatively to female patients [21]. Our results are, however, contrary to other population-based studies where female diabetic patients [2,7,8,15] and especially relatively young women [1,5,6] are often seen to have greater excess mortality than men. To a certain degree our results may be explained by the observed high general mortality of women relative to men when Denmark is compared with other Western-European countries . As can be seen from Table 1, it does not seem that our female patients in comparison with men have less severe diabetes or lower risk factor levels at diagnosis.

The relatively small number of deaths in people under 50 and over 80 years gives the estimated hazard rates and survival probabilities in these age groups wide confidence intervals (Table 3, Fig. 2 and 3). Although mortality in the general Danish population decreased during the period of the study, our results demonstrate that the excess mortality is highest among the youngest patients and decreases with increasing age in both sexes (Table 3). This is in accordance with most previous studies [1-3,6,8,9] including a Danish diabetes register based study [21] recognising this study included both type 1 and type 2 diabetic patients and probably did not encompass all newly diagnosed patients in Denmark. Even though our cohort of patients were recruited in 1989-92, and the mortality gap between diabetic patients and the general population may have lessened in recent years it does not seem to have disappeared [13,21]. As can be seen in figure 1, mortality rates are relatively constant over time for men and women in the three age groups. Therefore, we think that the finding of a lower relative risk of dying among the higher age groups compared to patients in the lower age groups is not due to a survivor effect, i.e. that surviving patients have a lower probability of dying, than newly diagnosed patients of the same age.

The excess mortality extends even to the old patients up to 80 years of age (Table 3, Fig. 2 and 3). Similar results were found in other studies with a large number of elderly diabetics [1,6,21] but our results are in contrast to other studies which showed no increased mortality for those diagnosed after 70-75 years of age [2,3,5,15]. These studies may reflect survival bias as pointed out earlier or too little power to show a real effect of age on mortality. Our results support that preventive treatment is advisable also for the old patients with respect for their personal values, motivation, capabilities and co-morbidities [28,29].

## Conclusion

We found that the diagnosis of diabetes gives an increased all-cause mortality risk in both men and women with type 2 diabetes, but men had a higher over-mortality than women. Although the excess risk of dying persisted in the old patients of both sexes it was most prominent in the youngest age groups.

## Competing interests

The authors declare that they have no competing interests.

## Authors' contributions

LJH wrote the research protocol, collected data for the study and drafted the manuscript. NO participated in the design of the study, was responsible for the inclusion of patients in 1989-92, and helped to draft the manuscript. VS performed the statistical analysis and helped to draft the manuscript. All authors read and approved the final manuscript.

## Pre-publication history

The pre-publication history for this paper can be accessed here:


